# Entering, Linked with the Sphinx: Lysophosphatidic Acids Everywhere, All at Once, in the Oral System and Cancer

**DOI:** 10.3390/ijms241210278

**Published:** 2023-06-17

**Authors:** D. Roselyn Cerutis, Michael D. Weston, Takanari Miyamoto

**Affiliations:** 1 Department of Oral Biology, Creighton University School of Dentistry, Omaha, NE 68178, USA; michaelweston@creighton.edu; 2 Department of Periodontics, Creighton University School of Dentistry, Omaha, NE 68178, USA; takanarimiyamoto@creighton.edu

**Keywords:** inflammation, lysophosphatidic acid, lysophosphatidic acid receptors, lysophosphatidic acid species, sphingosine-1-phosphate, GPCR, cancer, autotaxin, bone biology, periodontal disease, MALDI-MSI

## Abstract

Oral health is crucial to overall health, and periodontal disease (PDD) is a chronic inflammatory disease. Over the past decade, PDD has been recognized as a significant contributor to systemic inflammation. Here, we relate our seminal work defining the role of lysophosphatidic acid (LPA) and its receptors (LPARs) in the oral system with findings and parallels relevant to cancer. We discuss the largely unexplored fine-tuning potential of LPA species for biological control of complex immune responses and suggest approaches for the areas where we believe more research should be undertaken to advance our understanding of signaling at the level of the cellular microenvironment in biological processes where LPA is a key player so we can better treat diseases such as PDD, cancer, and emerging diseases.

## 1. Introduction

The mouth is the entrance portal to the body. Oral health is integral to general health, and some chronic diseases show a bidirectional association (reviewed in [1,2]); medical professionals often have limited knowledge of this connection as few programs teach oral health [3,4]. Some of these associations include chronic oral infections and diabetes, heart and lung conditions, some adverse pregnancy outcomes, and osteoporosis [5]. Numerous systemic infectious diseases, endocrine/metabolic diseases, and malignancies with underlying inflammatory bases, such as human papillomavirus infection/lesions, Type 2 diabetes (T2D), acute leukemia, and non-Hodgkin lymphoma, present oral and maxillofacial manifestations and may be identified by dentists by their clinical and radiological observations (reviewed in [6]).

Periodontal disease (PDD) is a chronic inflammatory disease attributable to an over-active host immune response to dysbiotic periodontal biofilm. Patients have individual genetic predispositions, lifestyle factors, and thus susceptibility profiles which affect their progression rate for this immuno-inflammatory disease and which determine the extent and severity of PDD that they develop. The incidence of PDD increases with age; it affects almost 50% of adults ≥30 years old, with approximately 70% of those 65 and older having varying degrees of PDD [7]. Over the past decade, PDD has been recognized to be a significant contributor to systemic inflammation (reviewed in [8,9]); increased inflammation levels are detrimental and predispose to the development of cancer. Michaud et al. (2018) [10] studied 7466 participants in the Atherosclerosis Risk in Communities (ARIC) study cohort (1996–1998) and found more evidence that for individuals with PDD, the risk for lung and colorectal cancer is especially elevated. This extended their previous findings that advanced PDD was associated with a 2.5-fold increase in smoking-related cancers among non-smoking subjects (19,933) and proposed that the immune dysregulation of PDD may be a predisposing factor [11]. Therefore, we will first attempt to weave the story of LPA in our oral system with findings and parallels relevant to cancer.

Chronic inflammation in PDD shares some features with the inflammation seen in cancer, particularly neutrophils and matrix metalloproteases [12,13], which has been called “the wound that does not heal”. While it is not the intent of this article to cover those, once the dysregulated, exaggerated immune response to the periodontal pathogens sets in, the inflammation in the periodontal tissue apparatus around the tooth becomes chronic—and without the appropriate treatment, it becomes “a wound that does not heal” until the periodontally involved tooth/teeth are successfully treated, extracted, or clinical attachment loss (CAL) and alveolar bone loss occur to the extent that they fall out. Once a tooth is absent, the inflammation-provoking entity is no longer there for the immune system to defend, so the inflamed periodontal tissue can then heal. This seems to be borne out by a study (of 51,529 men,1986–2012) that found a 31% higher risk of non-Hodgkin lymphoma (NHL) among participants with severe baseline PDD—but the risk was inversely associated with NHL after tooth loss [14].

### 1.1. LPA/LPARs

**Figure 1:**
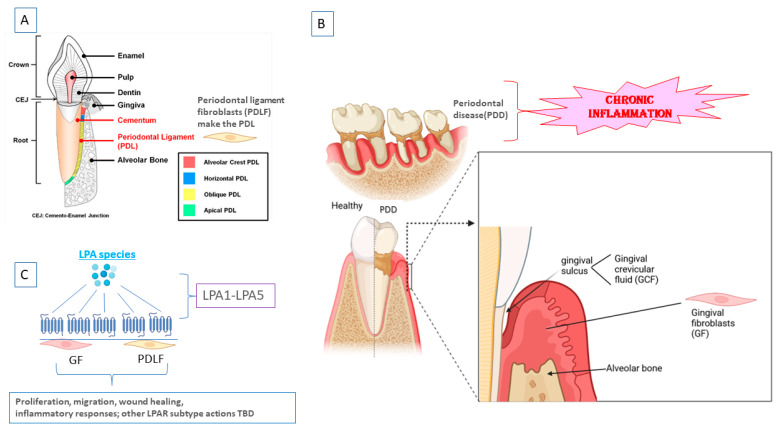
(**A**) Anatomy of the periodontal apparatus * (**B**) Teeth and the periodontal apparatus in health and in periodontal disease (PDD), showing the gingival sulcus (much smaller in health) from which the actively pumped gingival crevicular fluid (GCF) is obtained. (**C**) Gingival fibroblasts (GF) and periodontal ligament fibroblasts (PDLF) express LPA1-LPA5, which suggests that these receptors play critical roles in oral fibroblast homeostasis and inflammatory responses. * (Modified from [19]).

Our laboratory has dedicated its investigations to determining the role of lysophosphatidic acid (LPA) and its receptors (LPARs)- of which six (LPA1-LPA6) have been cloned—in oral homeostasis and in PDD (Figure 1). LPA is structurally the simplest phospholipid and is so biologically important that it is conserved down to the slime mold *Dictyostelium*. In mammals, it is central for embryological development, homeostasis, and in pathophysiology, particularly inflammation. It is found at low levels in most normal bodily fluids, but in pathology, it increases to pharmacologic levels and contributes to the disease processes in many systems. Virtually all somatic and immune cell types studied so far express multiple LPA receptors (LPARs) (reviewed in [15,16]). Most relevant for oral biology, LPA controls fibroblast activation, proliferation, and migration and promotes normal wound healing and collagen deposition [17]. LPA is also essential to regulating many key aspects of physiology and pathophysiology, which also govern oral tissues, including bone biology, epithelial barrier integrity, and inflammation (reviewed in [18]).

When we first set out to determine the significance of LPA in the oral system, one of the first homeostatic aspects we were interested in for LPA was investigating its involvement in oral wound healing, as it was shown that LPA significantly mobilized calcium and initiated cytoskeletal remodeling within minutes, while simultaneously increasing actin and focal adhesions [20,21,22]. Additionally, cellular migration was significantly enhanced by LPA, with both responses sensitive to pertussis toxin (PTX), implicating PTX-sensitive LPARs. Tomar et al. (2009) [23] showed that interaction of the actin-binding protein villin with LPA could dramatically alter actin reorganization outcomes as well as phospholipid-regulated cell signaling—and so LPA could function as an actin cytoskeleton intracellular regulator.

Dittmar and Hass (2023) [24] examined cancer cell fusion, a pathological process that increases malignancy and lowers survival; as with most tumors, metastases are the main cause of death in >90% of cancer cases [25,26]. Therefore, especially if cancer cells fuse with mesenchymal stem cells (MSC) and there is a subsequent post-hybrid selection process (PHSP), this significantly boosts metastatic ability [27,28]. Several authors have suggested that F-actin polymerization and associated cytoskeletal proteins play a vital role in allowing breast cancer cells to fuse with MSC within the tumor microenvironment [29,30]. This fusion process could be inhibited with cytochalasin D, an agent that blocks actin filament elongation, and suggests that the cytoskeleton and F-actin are essential to this cancer cell fusion process.

Therefore, LPA is of great interest as an essential regulator of the cytoskeleton and, by extension, of movement and migration, processes requisite to both wound healing and cancer cell fusion/metastasis.

Seminal in vitro work from our group [31,32] established that LPA is a key regulatory factor for primary human gingival and periodontal ligament fibroblasts (GF and PDLF) and, thus, for oral biology. We then showed that LPA positively regulates their wound-healing and regenerative responses [33] by signaling mainly through LPA1 and LPA3, which these fibroblasts express at high levels, and that they also express at least five (LPA1-LPA5) of the six cloned LPA receptors (LPARs) [34]. LPA1 appears to be the most active in LPA signaling [35]. It couples with G_i/o_, G_q/11_, and G_12/13_ to initiate downstream signaling cascades through phospholipase C (PLC), MAPK, Akt, and Rho. LPA1 regulates many cellular responses, including survival, proliferation, cytoskeletal changes, migration, cell–cell contact, intracellular Ca^2+^ mobilization, and inhibition of adenylyl cyclase (reviewed in [36]).

Critically, LPA is present where there is inflammation and/or bleeding, as blood platelets are the main source [21]. While our laboratory has focused on elucidating the role of LPA in oral homeostasis and in PDD, similar to LPA, S1P is also liberated from activated platelets [37]. Both LPA and S1P influence a multitude of basic cellular functions that include survival, proliferation, migration, and contraction. Therefore, many of the cellular processes regulated by LPA and S1P are fundamentally involved in wound-healing responses. Inflammation is an early component of wound healing; S1P-induced inflammatory response gene expression appears to be mediated through S1P1 and S1P3 and for LPA through LPA1 and LPA3 [35,38].

In contrast to Hashimura et al. (2020) [39], our data [40] showed that LPA levels become elevated from normal nM to µM (pharmacologic) levels in gingival crevicular fluid ((GCF), the actively pumped fluid that fills the gingival crevice at the base of a tooth) and in saliva from PDD patients. Given the extent of LPA’s regulatory actions on oral fibroblasts, we hypothesized that LPA would control multiple transcripts related to wound healing and inflammation and designed a microarray experiment based on stimulation of primary GF (n = 3; three pools of three healthy young donors each) stimulated with the most widely used LPA species, 18:1, and explored the results by Ingenuity Pathway Analysis (IPA) of molecular interaction pathways. LPA exerted profound transcriptional control over >60 key GF inflammation-related cytokines, their receptors, enzymes, and other mediators [41,42]. Other investigators [43,44] have reported that LPA and its receptors control the biology of human dental pulp fibroblasts and of human oral keratinocytes, respectively. Further, Kim et al. (2020) [45] showed that in human periodontal ligament stem cells treated with *P. gingivalis* lipopolysaccharide to mimic the inflammation seen in PDD, the LPA1 antagonist AM095 lowered their expression of pro-inflammatory cytokines and promoted osteogenic differentiation. Their results further support our finding of the importance of LPA1 for oral cells. The totality of these studies has unequivocally established that LPA is an essential mediator in the oral system.

In dentistry, bone remodeling/healing after oral surgeries and dental implant placements is paramount [46]. LPA is required in normal bone biology, as LPA1 promotes, while LPA4 inhibits osteoblast differentiation [47]. The LPA-LPA1 axis is indispensable for bone homeostasis, as LPA1 null mice show major defects in osteoblast and osteoclast activity [48]. Chen et al. (2019) [49] reported that LPA enhanced the alkaline phosphatase and matrix mineralization activity of pre-osteoblastic cells and that both of these effects were reversible by pharmacologic blockade with the dual LPA1/LPA3 inhibitor Ki16425.

### 1.2. Autotaxin

Autotaxin (ATX), the main LPA-synthesizing enzyme, is unique in that it is a secreted lysophospholipase D that uses plasma membrane phospholipids and circulating lysophosphatidylcholine (LPC) bound to albumin to generate LPA. We showed that human GF make ATX (ENPP2) transcripts and that they also produce several LPA species in a time-dependent manner [42], as do human PDLF. Of great interest for PDD, which has a genetic susceptibility component, we saw a marked induction of ATX/ENPP2 in the third donor group of our microarray survey at both 2 h and 8 h vs. the small but significant changes in the first and second groups at 2 h and the decreases (not significant) at 8 h for these two groups [41,42]. The enhanced response in the third group was likely due to a donor with a genetically over-responsive immune system, which would predispose them to develop PDD at a later age. With at least two periodontal cell types (GF and PDLF) capable of synthesizing LPA locally, we have proposed it then acts in both a paracrine and autocrine fashion. We base this on the fact that in order to determine LPA’s actions on oral fibroblasts, as is routine, we serum-starve them overnight to mitigate any stimulation from the µM LPA present in fetal bovine serum. When we tested longer periods of total serum deprivation (up to five days), both human GF and PDLF (from multiple donors) survived without going apoptotic; this result has never been observed by D.R.C. with any other cell type but is fully in keeping with reports that LPA exerts anti-apoptotic activity in most cell types (reviewed in [18]). So, LPA was very likely acting in an autocrine/paracrine fashion in these oral fibroblasts to prevent their apoptosis.

As alveolar bone integrity is crucial for a healthy periodontal apparatus, we hypothesized that ATX likely contributes significantly to the pathogenesis of PDD. ATX is essential to blood vessel development, so ATX-deficient mice exhibit severe vascular defects and die around E10.5 [50,51]. Heterozygous ATX-null mice have serum ATX levels that are approximately half that of wild-type mice, so LPA can still be produced at levels that would confound the interpretation of results in a mouse model of PDD. Regeneron Pharmaceuticals made an inducible ATX knockout (R26Cre-ER^T2^ mice (line 2151)); however, inducing the Cre recombinase requires using intraperitoneal injections of Tamoxifen for 10 days [52]. In our opinion, tamoxifen use for testing the role of ATX in our PDD model is problematic, as steroid effects are long-lasting; critically, as LPA3 is involved in embryo implantation and spacing, it is steroid-sensitive [53,54].

Periodontal tissues are also very steroid-sensitive/dependent; gingival recession begins at menopause ([55], reviewed in [56]). Thus, we addressed that knowledge gap by administering the potent, orally bio-available ATX inhibitor PF-8380 (IC_50_ 1.9 nM) by gavage in a mouse model of PDD induced by oral infection with *P. gingivalis* (strain FDC 381) and have found approximately 40% reduction in alveolar bone loss (study ongoing), so it appears that ATX does play a significant role in the pathogenesis of PDD.

The ATX-LPAR axis is also a major contributing factor in the progression of numerous cancers, many of which originate in or metastasize to bone. The inflammatory interleukins-6 and -8 (IL-6 and IL-8) produced in vitro by LPA-stimulated oral squamous cell carcinoma (OSCC) promoted osteoclastogenesis and bone resorption [57]. In adult mice, ATX is expressed in high endothelial venules (HEVs) as well as in some blood vessels of chronically inflamed tissues [58]. In our human GF system, LPA exerted highly significant transcriptional control over >60 key inflammation-related cytokines, their receptors, enzymes, and other mediators, including IL-8, IL-11, and COX-2 [42].

### 1.3. Sphingosine-1-Phosphate (S1P)

S1P is a small signaling lipid involved in controlling a multitude of biological processes and serves as a master regulator of immune cell activation and trafficking and of cytokine secretion ([59], reviewed in [60]). Its chemical backbone is sphingosine, and it has a single, covalently bound fatty acid (FA). It is made from sphingosine by two enzyme isoforms, sphingosine kinase-1 and sphingosine kinase-2 (SPHK1 and SPHK2). Responses to S1P are determined by the cellular compartmentalization of the enzymes, and the complement of S1PRs expressed, as well as by the SPHK isozyme(s) expressed.

SPHK is a pivotal enzyme in homeostasis and oncogenesis and is further complicated by the emerging picture that each SPHK isozyme also produces alternately spliced isoforms, which affect cancer resistance. This is very significant to treatment outcomes, which appear to also be influenced by the SPHK isoform expressed (reviewed in [61]).

The sources of serum S1P are mainly platelets, white blood cells (WBC), and endothelial cells, although most cells produce S1P as part of normal sphingolipid metabolism [62,63]. The median normal (healthy) S1P serum concentration was found to be 0.804 µmol/L in the Study of Health in Pomerania (SHIP-Trend) cohort [64,65]. The bulk of S1P is transported bound to apolipoprotein M (apoM), with a much smaller percentage (30%) bound to albumin [62,63], which by contrast, serves as the major carrier for LPA.

Moritz et al. (2021) [66] reported that in human PDD, subjects showed elevated serum S1P levels, and inflamed gingival tissue demonstrated significantly increased SPHK1, with intense staining in epithelial and CD68+ cells. Our primary human microarray data of LPA-treated GF showed stimulation of SPHK1 mRNA in all three groups (3.9 ± 0.5- and 4.7 ± 0.7-fold at 2 h and 8 h, respectively [42]). This differed substantially from the responses of primary human foreskin fibroblasts (≤passage 10), where the fold changes were reported to be 0.21- and 0.52-fold at 1 and 8 h, respectively [67]; this difference is attributable to GF’s enhanced inflammatory capacity stemming from their constant exposure to the complex oral microbiota, predominantly commensals in health and a dysbiotic microbiota shifted by periodontal pathogens in PDD.

Given that Moritz et al. (2021) [66] found elevated serum S1P levels in PDD patients, we speculate that our observed marked induction of ATX/ENPP2 in the third donor group of our microarray survey at both 2 h and 8 h vs. groups one and two [42] would likely lead to a parallel increase in both localized and serum LPA from the increased ATX activity in genetically PDD-susceptible patients, of which there was likely at least one donor in group three—but not in the first two groups that showed low responses. For inflammation, S1P is among the most potent mediators that switch bone marrow macrophages (BMMs) to the classical pro-inflammatory M1 phenotype [68]. It has been reported that S1P2 and S1P3, but not S1P1, mediate the S1P-induced BMM polarization to the M1 phenotype in vitro [69]; M1 macrophages are positive for a specific marker, CD86, and also produce pro-inflammatory cytokines such as IL-1, IL-6, tumor necrosis factor (TNF)-α, and macrophage inflammatory protein (MIP)-1β/CCL4, among others.

When S1P concentrations were measured in bone marrow, they were lower than in plasma [70]; S1P appears to dynamically regulate the in vivo migration and regulation of osteoclast precursors. Given the strong induction of SPHK1 message in GF by LPA [42], which presumably would generate S1P locally, we maintain that it would serve to recruit osteoclast precursors to degrade alveolar bone in PDD by increased local S1P gradients. Similar to LPA, which is known to be a major player in the establishment and progression of joint damage in rheumatoid arthritis (RA), S1P is a key mediator in this process as well (reviewed in [71,72]).

The induction of SPHK1 by LPA in our oral system is made more significant given that in PDD, S1P has been found to induce pro-inflammatory cytokine production: interferon β and IL-6 and IL-8 in primary human gingival epithelial cells via S1P1 and S1P3 [73] and in a mouse model of *Aggregatibacter actinomycetemcomitans*-induced PDD, periodontal inflammation and alveolar bone loss were attenuated in SPHK1-deficient animals [74]. Therefore, we suggest that the elevated LPA levels we found [40] and S1P are both contributing to the inflammatory loss of attachment and alveolar bone loss characteristic of PDD.

LPA is an indispensable regulator of the actin cytoskeleton and focal adhesion formation [21]. A compelling discovery with implications for our PDD system and for cancer is that LPA regulated the actin-binding protein gelsolin (which also binds it) in a manner similar to phosphatidylinositol 4,5 bisphosphate (PI(4,5)P2, or PIP2). It dissociated the complex formed between actin and fragminP, unlike other lysophospholipids or S1P, which were both inactive. Furthermore, LPA inhibited the F-actin severing activity of human gelsolin. Significantly, LPA promoted gelsolin release from barbed actin filaments (the “barb” is the end at which monomer addition preferentially occurs for control of filament assembly) in permeabilized human platelets-suggesting that LPA can act intracellularly to modulate actin-binding proteins [75].

In a greatly significant finding, screening research showed gelsolin downregulation in most tumors—and differential expression in differing molecular and immunological cancer types. Serum gelsolin showed varying impacts on tumor type prognosis. However, the prognostic efficiency was moderate to high, with serum gelsolin concentration showing good diagnostic value for breast cancer (as a common example). Furthermore, gelsolin was a differentiating prognostic factor for certain specific types of cancers. Most cancers showed hypophosphorylated gelsolin, and in most cancers, the gelsolin promoter was found to be hypermethylated. When analyzing tumor-infiltrating immune cells, gelsolin was linked to the level of infiltration seen and indispensable for those immune cells to infiltrate the tumors. KEGG (Kyoto Encyclopedia of Genes and Genomes) and Gene Set Enrichment Analysis (GSEA) analyses showed gelsolin to be vital in cancers for DNA methylation, cell cycle, functions of proteoglycan pathways, chemokine signaling, and immune-related pathways. The authors proposed that gelsolin has the properties to be a pan-cancer diagnostic, predictive, and immune indicator [76].

## 2. Our Perspective: Major Knowledge Gaps in the LPL Field for Investigation

After many decades of cancer research, we are now able to successfully treat some cancers, while many more still remain inconsolably incurable and cause much societal suffering and a huge economic burden.

**Figure 2:**
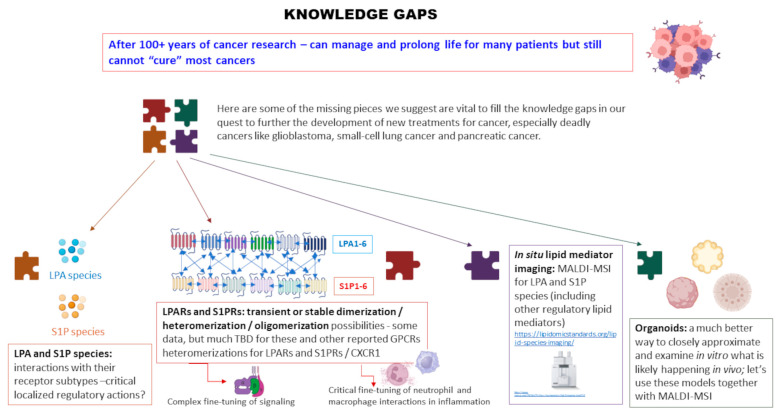
Our Perspective: Major Knowledge Gaps in the LPL Field where we believe that more investigations should be pursued. Abbreviations: CXCR1, C-X-C motif chemokine receptor 1, a receptor for interleukin 8 (IL-8). It binds to IL-8 with high affinity and transduces the signal through a G-protein-activated second messenger system. R, receptor. MALDI-MSI, matrix-assisted laser desorption/ionization (MALDI) mass spectrometry imaging (MSI), a powerful analytical platform for tissue diagnostics.

In their recent excellent and incisive editorial [77], Drs. Thorp and Yaffe point out that we are drowning in data but have not reaped most of the predicted expected benefits. They argue that there is “much we fundamentally still do not understand” and that “Fundamental science, pursued rigorously, has never mattered more”. Indeed. While their commentary focus is on analyzing the “result of applying advanced machine learning methods to biological science”, we agree with their timely reminder that a rigorous understanding of mechanisms and pathways is the only way forward to ultimately deeply understand cellular signaling in biological processes so we can prevent and/or treat existing and new diseases such as PDD, cancer, and emerging diseases (see Figure 2).

### 2.1. LPA and S1P Species

The widespread expression of LPARs and their varied cohorts and expression levels in different cell types and tissues means that increased systemic/local LPA production, or decreased LPA degradation, can affect many physiological processes and organs [78,79].

LPA exists as multiple molecular species with a fatty acid of different chain lengths and degrees of unsaturation covalently bonded to the glycerol backbone in an acyl, alkyl, or alkenyl linkage; it has been reported for some systems that different LPA species exhibit differing affinities for certain of the cloned LPAR subtypes [80,81,82]. Ray et al. (2020) [83] recently used a highly sensitive assay combining a free-solution assay (FSA) with compensated interferometric reader (CIR) to quantify native binding interactions between LPA1 and multiple LPA species in free solution without labeling. They reported the following (K_D_ ± SEM, nM): 18:2, 2.83 ± 1.64; 20:4, 2.59 ± 0.481; 16:0, 1.69 ± 0.1; no K_D_ values had been previously reported for these species. Their results for LPA 18:1 were 2.08 ± 1.32 nM, while Mizuno et al. (2019) [84] used backscattering interferometry and reported 0.87 ± 0.37 nM, and (Yanagida et al. (2009) [85] found the K_D_ to be 68.9 nM using radioligand binding with [^3^H]-1-oleoyl-LPA. As more investigators come forward with additional binding data acquired under near-native conditions, we will move closer to determining which of these methods more closely reflects the in vivo affinity of individual LPA species for different LPARs.

In a manner analogous to LPA, the FA side chain of the S1P ceramide is also variable, thus yielding a multitude of unique species as well, with those having different bio-activities and actions in different cell types (reviewed in [86]).

Studies utilizing multiple LPA and/or S1P species are few, presenting a very large gap in our understanding of what critical localized regulatory actions these species exert in vivo, given the multitude of possibilities that stem from their possible respective interactions with the five LPARs and five S1PRs that have been cloned to date. The caution of Leblanc and Peyruchaud (2015) [87] that “Such considerations deserve attention when interpreting in vitro studies because they are predominantly carried out using 1-acyl-LPA (18:1)” certainly applies.

From our PDD research experience and perspective, we believe that as PDD and cancer are both inflammation-based diseases and LPA and S1P are so key in cancer development, progression, and metastasis to bone (for those that do) that their species’ signaling interactions must be determined and finely dissected, as significant differences are very likely to emerge for each oral cell and cancer cell/type. Understanding these differences will help us to design more targeted treatments for PDD, as well as cancer treatments with less serious side effects, which will help save/prolong lives.

Sugiura et al. (2002) [88] reported that normal human saliva contains ~0.785 nmol/mL LPA, with the predominant species being LPA 18:1 > 18:0 > 16:0. So when we went to determine the effects of LPA on regenerative and intracellular calcium responses in multiple isolates of primary human GF and PDLF from healthy young donors [33], we were surprised to find minimal responses to 18:1 for PDLF, as it was reportedly the main salivary species. Instead, the 16:0 and 18:0 species most robustly significantly stimulated [Ca^2+^]_i_ in PDLF compared to GF, which by contrast, responded significantly to all three of these salivary species. These pertussis toxin (PTX)-sensitive responses were antagonized by Ki16425, a dual LPA1/3 antagonist. Furthermore, when GF and PDLF chemotactic responses were stimulated in this study using the subtype-specific agonists NAEPA (LPA1), FAP-12 (LPA2), and 2S-OMPT (LPA3), we unexpectedly found that GFs seemed to be responding via LPA1, whereas PDLF migration was stimulated through LPA1, LPA2, and LPA3. Later, we determined that primary GF and PDLF also produce these LPA species in a time-dependent manner [42].

LPA species are also variable in cancer. Chae et al. (2022) [89] identified that for ovarian cancer (OvCa) patients, ten different LPA species were detected in their ascites fluid, with the top three being LPA 16:0 > 18:0 > 18:2; this was mirrored in ascites from their OvCa mouse model. They also found that LPA quickly induced Ptgs2 (Cox-2) production by the bone marrow dendritic cells in a time- and dose-dependent manner. This led to PGE_2_ overproduction and suppression of anti-cancer immunity (via multiple mechanisms, as previously shown) [90,91]. However, only the 16:0 and 18:2 species were significantly positively associated with PGE_2_ expression and correlated with decreased survival in the high-grade serous OvCa patients. Reinartz et al. (2018) [92] found that all major acyl LPA species (16:0, 18:2, and 20:4) are present in ascites from high-grade serous adenocarcinoma (HGSC) and contribute to promoting HGSC motility and invasion. They also found that tumor-associated macrophages (TAMs) were the main producers of ATX and 20:4 acyl-LPA, with the latter not produced by tumor cells.

Dacheux et al. (2022) [93] found that in a mouse model of melanoma metastasis to the lung, the predominant plasma LPA species were 16:0 > 18:2 > 18:0. Turner et al. (2023) [94] just published a study examining the role of LPA in CD8 T cell metabolism and immunosurveillance in the melanoma system. They found that CD8 T cells have important tumor-fighting functions that are impaired by LPA via LPA5, which reprograms these cells to promote an “exhaustion-like state, both in vitro and in vivo”. LPA changed mitochondrial respiration in these cells, leading to increased fatty acid oxidation and proton leak; maximal respiration and proton leak, but not basal respiration, was mediated through LPA5 and could be rescued by Lpar5 deficiency or LPA5 antagonism. While they also found that B cells, macrophages, and NK cells also express LPA5, they noted that the actual roles and function of LPA5 on myeloid cells are only poorly understood to date and encouraged more researchers to pursue solving these questions. In their stage 4 patients, LPA 16:0 and 18:1 were the most abundant of the species measured, and plasma LPA levels predicted survival. Those with lower concentrations of LPA 16:0 responded better to immunotherapy than those with higher levels, and this was the only LPA species found to be significantly different between responders and non-responders.

Head and neck squamous cell carcinoma (HNSCC) is a worldwide problem and has a poor prognosis. Using targeted lipidomics with liquid chromatography triple quadrupole mass spectrometry (LCMS/MS), the concentration of LPA (16:0, 18:0, 18:1, 18:2, and 20:4) in plasma from patients with oral squamous cell carcinoma (OSCC) and nasopharyngeal carcinoma (NPC) and healthy controls was analyzed; three LPA species (18:1, 18:2, and 20:4) were significantly lower in OSCC patients, and all five LPA species were significantly lower in NPC plasma. However, the order of abundance of LPA species differed between the control and cancer groups, with LPA 16:0 and 18:0 more abundant in OSCC and NPC patients [95]. Of interest, our studies confirm LPA (18:1) induction of Ptgs2 (Cox-2). In our human GF system, it was one of the top regulated transcripts (2.4- to 35.5-fold), so we proposed [42] that the LPA over-production seen in PDD saliva and GCF [40] would serve to fuel inflammation and worsen the condition. We have just confirmed that in our mouse model of *P. gingivalis-*induced PDD, there is the same 10-fold elevation of LPA species in mouse saliva [96] as we have reported for human saliva [40]. In vivo, LPA circulates bound to albumin in human plasma, although it also binds to the actin-severing protein gelsolin with an affinity (K_D_ = 6 nm) similar to that of LPA for LPA1, LPA2, and LPA3 and greater than that of serum albumin (K_D_ = 360 nm) [89]; the authors speculated that serves to differentially deliver LPA more efficiently to cells that produce gelsolin, such as myocytes [97], which also make S1P and LPA, both which stimulate [Ca^2+^]_i_ release to stimulate myocyte contraction [98].

However, gelsolin also protects against oxidative stress, inflammation induced by microbes, and the toxicity of free actin released by damaged cells (reviewed in [99]). Of great significance for cancer, gelsolin is essential for anti-tumor immune cell infiltration (we refer the interested reader to a recent review [100]).

As with other investigators in the LPL field, we use Fraction V fatty acid-free bovine serum albumin (FAF-BSA) to make all our LPA dilutions for our human oral cell-based experiments. However, a study has been published that is a cautionary tale—the confounding that may result from extrapolating from in vitro studies using “unnatural” conditions, such as utilizing proteins from a different species than the test cells. Fleming et al. (2016) [101] used a novel technique utilizing monoclonal anti-LPA and anti-S1P antibodies with a Kinetic Exclusion Assay to measure the equilibrium dissociation constants (K_d_) for the carrier proteins binding LPA [FAF-BSA and fatty acid-free human albumin (FAF-HSA)] and apoM-HDL and apoM-LDL for S1P. They found that FAF-BSA bound LPA 16:0, 18:1, 18:2, and 20:4 with the following K_d_s, respectively: 68 nM, 130 nM, 350 nM, and 2.2 µM; for S1P, it was 41 µM. By contrast, FAF-HSA bound each LPL with comparable affinities. The authors noted that this study provided insights into LPA and S1P storage in circulation. We agree and caution that investigators should be aware of these differences for experimental design and for data interpretation, as this has real implications for LPAR and S1PR activation and signaling.

Galvani et al. (2015) [102] and Blaho et al. (2015) [103] both published data indicating that S1P can exert different biological activities depending on the chaperone protein it is bound to. Wilkerson et al. (2012) [104] demonstrated that S1P bound to apoM-HDL sustained human endothelial cell barrier function longer than FAF-BSA-bound S1P. This also affected the kinetics of S1P1 internalization, as when bound to FAF-BSA, the cells internalized and degraded the receptor faster.

**Figure 3:**
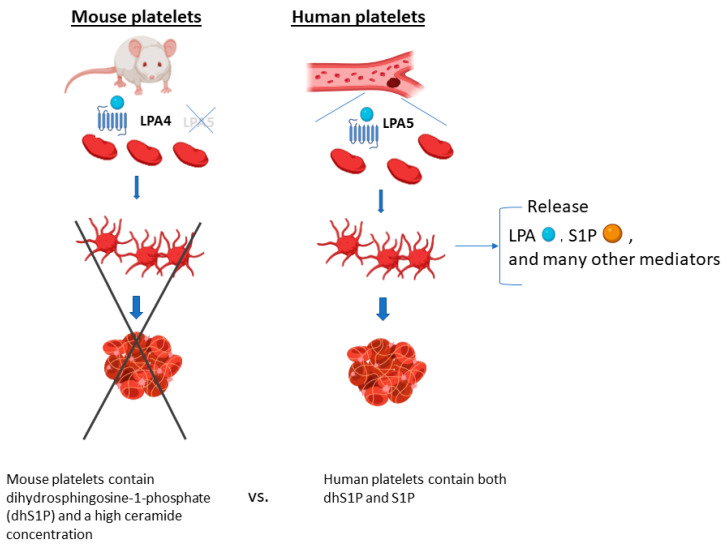
Differences between mice and humans in platelet LPAR expression/sphingolipids. Cancer, inflammation, and thrombosis are interrelated, with platelets being a common shared element. Human platelets express LPA5, while mice do not—thus, LPA does not trigger platelet activation and clot formation in mice. LPA and S1P are co-liberated from activated platelets, while non-activated mouse platelets contain dihydrosphingosine-1-phosphate (dhS1P) together with a high ceramide concentration, whereas human platelets contain both dhS1P and S1P [105].

A major caution for interpreting studies involving platelets or LPA/sphingolipids using mouse models (Figure 3) is that human blood sphingolipid distribution is very different than that of mice, and their platelets differ in LPAR expression. LPA and S1P are co-liberated from activated platelets, and non-activated mouse platelets contain dihydrosphingosine-1-phosphate (dhS1P) together with a high ceramide concentration, whereas human platelets contain both dhS1P and S1P [105].

Given the species differences, we now speculate that had we had this information and so used FAF-HSA instead of FAF-BSA as an LPA carrier in our GF and PDLF experiments, our experimental results may have been different—and that possibility should be tested.

### 2.2. In Situ Lipid Mediator Imaging

Lipids have been demonstrated to serve as master regulators, especially LPA and S1P. Many investigators, including those in our laboratory, have measured LPA and/or S1P levels in the circulation and in most bodily fluids ([40,65], reviewed in [18]). At the cellular level, glycerophospholipids and sphingolipids change dynamically both at the plasma membrane and nuclear membrane [106]. Obtaining localized spatial information at the micro-environment at the cells’ level is what will ultimately help us to understand the local interactions that will ultimately determine disease development and progression. Therefore, we suggest that imaging mass spectrometry is a tool that should be applied much more going forward to give us localized spatial information as to where the individual LPA and S1P species are found in inflammation-based diseases such as PDD and cancer and to map how these mediators’ distributions change throughout disease/malignancy development, progression, and metastasis.

The powerful technique of matrix-assisted laser desorption ionization-mass spectrometry imaging (MALDI-MSI) allows for label-free imaging of molecules. This includes localizing more abundant phospholipids in tissue sections at microscopic resolution. Recently, a study undertook the investigation of neurolipid (endocannabinoid), LPA, and S1P signaling and specific lipid species in a mouse model of Alzheimer’s disease (AD) using MALDI-MSI for lipid localization and quantitation and [^35^S]GTPγS autoradiography to locate the cannabinoid CB1, LPA1, and S1P1 receptor subtypes [107]. Their elegant work pioneered the anatomical localization of lipid species in a mouse brain model of AD and was able to report changes in both lipid composition in different brain regions and in S1P1-mediated signaling. However, they were unable to detect ceramides with their experimental conditions [108], which prevented them from doing a more complete analysis of brain sphingolipid metabolic turnover in this model.

As noted by Gonzalez de San Roman et al. (2021) [107], imaging sphingolipids has been challenging. For LPA, it can be particularly problematic, as it can be produced as a methodological artifice from other phospholipids fragmenting during traditional MALDI-MS analysis. This phenomenon is known as an “in source decay” (ISD) fragmentation [109]. LPA and S1P are key molecules in so many homeostatic and pathological processes and conditions that the Lipidomics Standards Initiative (LSI; https://lipidomics-standards-initiative.org/, accessed on 3 May 2023) is actively working towards improving the sensitivity of detection for LPA, S1P, and related lipids [110].

Phos-tag is a derivatization reagent which is a zinc complex that specifically binds to a phosphate mono-ester [111]. Morishige et al. (2010) [112] optimized the matrix and on-tissue derivatization protocol to improve upon a previous Phos-tag method which allowed for the detection of monocationic complexes with LPA and S1P by liquid chromatography (LC)-MALDI-MS, [. Their optimized methodology allowed visualization of mouse brain LPA species and S1P distribution and localization with accuracy and high sensitivity [113]. Of particular note, this group found S1P to be undetectable without Phos-tag derivatization. Their cross-check results using laser microdissection (LMD) LC-MS/LMD agreed with the results found for LPA by the Phos-tag method, which they stated proved that the distribution and amounts of LPA and S1P detected with their improved method were correct and not due to artificial ISD fragmentation. This methodological advance now gives the LPL field a powerful discovery tool.

### 2.3. GPCR Multimerization and Signaling Implications

Alekseenko et al. (2023) [114] convey the frustration and limited advancement in treatments produced to treat cancers by targeting molecular entities; they focus on the “…the Sparkling Hope of Supramolecular Targeted Strategies” to give us the cures we need. They extensively cover the immensely complex interactions between cancerous and immune cells (likely through “synapse” formation between multiple cell types, including mesenchymal stem cells (MSCs), cancer cells, and immune cells); GPCRs are immensely important in this process as they are critical components in many of these “synapses”. By directly physically associating (henceforth referred to as “multimerization”), GPCRs can affect each other’s function and thus elicit different cellular responses through altered signaling and/or trafficking patterns. While this work does not purport to be an exhaustive review of GPCR multimerization, we will cover some of the latest, most physiologic findings in near-native (cell culture at 37 °C) or native conditions, as well as technique-related advancements in the field that we feel hold much promise to help reveal the actual workings of these complex associations.

Although receptor dimerization was first noted four decades ago [115,116,117], it was at first controversial, and its critics attributed the findings to experimental artifacts. Vischer et al., (2015) [118] have reviewed the developments that had to take place to study GPCR multimer stability and stoichiometry and to detect GPCR dimers under native conditions. Now, many more GPCRs have since been reported to form transient or stable homo- or hetero-/dimers/oligomers, and their in vivo existence has been confirmed using native tissues [119,120]. Using a time-resolved fluorescence resonance energy transfer (TR-FRET)-based approach, Albizu et al., (2010) [119] used patches of lactating rat mammary gland to demonstrate oligomerization for the vasopressin (AVP) and oxytocin receptors and also the dopamine D2 receptor. In their system, excess agonist or antagonist addition did not destabilize dimers, and antagonists did not promote dimer formation. FRET efficacy between agonists and antagonists for the D2 receptor was very similar to that for the vasopressin receptor, showing that these receptor associations happen in more than just one GPCR family. Kasai et al. (2018) [121] used single-molecule tracking in physiologic conditions (cell culture system at 37 °C) to determine homodimer lifetimes (in milliseconds) of the D2 receptor before and after the addition of agonist or neutral antagonist. They found that before ligand addition, it was 68.4 ± 4.8 ms; after adding 15 µM dopamine, it was 98.6 ± 8.3 ms; after adding the agonist quinpirole at 15 µM, it was 103.5 ± 9.3 ms; and after adding the neutral antagonist UH-232 at 0.1 µM, it was found to be 70.5 ± 11.0 ms. These results supported observations that acute amphetamine exposure enhanced rat brain D2 receptor dimerization without affecting its expression levels [122].

Rivero-Müller et al. (2010) [120] elegantly used transgenic mice co-expressing binding-deficient and signaling-deficient forms of the luteinizing hormone receptor (LHR) in the Leydig cells of the testes to reconstitute normal LH hormone action through intermolecular functional complementation in the absence of wild-type LHRs, thus proving intermolecular cooperation upon LHR activation.

Liu et al. (2022) [123] reported biased signaling of the platelet-activating factor receptor (PAFR). They used human HEK-293 cells transfected at low density with the human PAFR (mCherry-fused Halo-PAFR) in single-molecule photobleaching studies and were able to visualize PAFR oligomer formation on the cell membrane. They found dimerization-induced biased signaling, which was also observed with a naturally occurring PAFR genetic variant.

Significantly for inflammation, another layer of complexity exists for these receptors: GPCRs, similar to LPARs and PAFRs, are also found in the nucleus [124]. A study linked LPARs’ interaction with other GPCRs to regulate inflammatory transcripts: the PAFR and LPA1 co-localize with caveolae at the nucleus, together with the prostaglandin EP2 receptor. This arrangement allows them to be ideally localized to regulate the inflammatory transcripts such as inducible nitric oxide synthase (iNOS) and COX-2 [124]. GPCR intracellular signaling has also been more recently confirmed by studies of the thyroid-stimulating hormone receptor (TSHR) [125]. Our laboratory has found LPA1-3 on intact human periodontal ligament and human gingival tissue nuclear membranes by confocal microscopy [126,127]); we posit that the findings of Zhu et al. (2006) [124] likely also apply to the PDD system.

So far, the Class A and B GPCRs tested can transduce agonist-induced intracellular signaling as monomers, while Class C GPCRs (such as the much-studied γ-amino butyric acid receptor (GABAbR) need to dimerize to signal in response to agonists (reviewed in [118]).

So, the biological question is why do Class A and B GPCRs form multimers? It has been postulated that multimers serve for specialized signaling, as reviewed in Milstein et al. (2022) [128], whose work catalogs the studies using single-molecule counting methods to quantitatively characterize the distribution of oligomeric assemblies of various GPCRs, several of which tracked the spatiotemporal oligomerization behavior of GPCRs in live cells. The reality of GPCR multimerization drives home that this biological phenomenon has a significant role in endogenous agonist signaling and in drug action, so having a complete understanding of this process is necessary for optimal drug design. Shonberg et al. (2011) [129] discuss the combinations of receptors that continued to be discovered to form homo- and heterodimers, as well as higher order oligomers in natural tissues, and cover the topic of bivalent ligands for GPCRs and their in vivo properties for drug design.

We determined by flow cytometry that human GF and PDLF express at least LPA1-LPA5 [34]. Their expression of so many LPAR subtypes supports and leads to the inescapable conclusion that LPA is a critical mediator necessary for these cells’ functions and that the actions of LPA must be exerted via at least these first five of the six cloned LPARs. From our studies to date [33], it appears that LPA1 and LPA3 are the main subtypes mediating human GF and PDLF regenerative responses, but we are still determining the functions of the other LPAR subtypes these cells express.

LPA1 has been shown to be a crucial receptor in many different types of cancers; we will not cover it here (the reader is referred to [130]). Of great interest, LPA1, S1P3, and S1P4 have been shown to form constitutive heterodimers with an IL-8 receptor, chemokine (C-X-C motif) receptor 1 (CXCR1), thus affecting the function of neutrophils [131] and by extension, when neutrophil behavior is altered, macrophage (MP) function is also affected as their functions in inflammation are intertwined (reviewed in [132]). They also found that LPA treatment reduced the amount of LPA1/CXCR1 heterodimer. These immune implications are equally important to the inflammation seen in PDD and cancer.

Crucially, LPA1 and LPA3 were found to form heteromers with S1PRs [133]. We also believe that the LPARs and S1PRs homo- and hetero-dimerize—and likely also form oligomers—to mediate their functions in both health and in PDD, as well as in cancer and other diseases. This is a hypothesis we have not been able to test, as that is not the focus of our laboratory but which now needs to be explored by other investigators.

S1P is the master regulator of leukocyte trafficking [60]. As the endothelium is critical to this complex process in health and in inflammation, the urgency of understanding the hetero/oligomerization of LPARs and S1PRs and its effects on their signaling interplay cannot be overstated. Indeed, the importance of dissecting the relationship of LPARs with S1PRs for inflammatory conditions such as PDD and cancer is exemplified by the work of Hisano et al. (2019) [134]. They found that in lymphatic endothelial cells (LECs), LPA1 affected the downstream signaling bias of S1PR; the induced inter-GPCR β-arrestin coupling attenuated S1P1-induced LEC barrier function, enhancing endothelial porosity. GPCRs are present at the plasma membrane in different conformations and in different multimeric states; the research progress made can be seen interactively at http://www.gpcr-hetnet.com/ [135]. However, our knowledge of GPCR-GPCR interactions is still in its infancy. To facilitate studying these GPCR interactions, newer approaches have been reported for investigating GPCR function in vivo under conditions that can be rigorously controlled. They use genetic mouse models with advanced fluorescence imaging to observe the availability of GPCR ligands, their activation, and signaling [136], which offer a promising avenue of investigation.

### 2.4. Organoid Models

With the ongoing development and refinement of complex organoid models containing many of the features of the tissue/organ they are aiming to replicate (reviewed in [137]), we now have within reach a much better way to closely approximate and examine in vitro what is likely happening in vivo. A limiting reality for most models has been the lack of proper microvascularization. Because of this obstacle in obtaining enough oxygen and nutrients along with waste product removal, most organoids have been unable to assume optimal structural complexity due to this limitation in forming the complex vascular network needed to mimic in vivo interactions between tissue and vascular system. However, together with 3D and 4D bio-printing, micro-flow control technology is emerging to address this need. In vivo vascularization can be achieved by transplanting organoids started in vitro into the desired host—and is so far the best way to achieve completely functional organoids, as the vascularization process proceeds as it normally occurs in the body, although there remain differences from the native organ’s blood vessels (reviewed in [138]). That said, the existence of these models offers a major step forward. We need this option, as animals have differences from humans in both metabolism and immune function, and using them in disease models has confounding implications [114,131,139], which have impeded us from advancing as far as we have wished. The interested reader is referred to Hoffman et al. (2022) [140] for a systematic review of clinical outcomes of organoid research, where they detail the clinical studies planned or already underway.

## 3. Major Pieces of a Vast and Complex Puzzle: Conclusions from Our Perspective

In the lysolipid field, researchers continue to dissect the signaling pathways for the cloned LPA and S1P receptors, and we now have a growing (if still imperfect) understanding of their in vivo functions; however, we have scant knowledge of how these receptors may interact in vivo once they homo-, hetero-, and/or oligo-merize, as these GPCRs have been reported to do [118]. How does that ultimately affect the endogenous ligand binding, signaling, and ultimate function of these multimeric entities? We need more attention to and studies in this area.

Add to that the complexity of the multiple species of both LPA and S1P that exist in vivo, and the permutation possibilities rapidly escalate the fine-tuning complexity for biological control of responses. The organism makes these species for a reason. Therefore, we need a solid understanding of the function of each of these species through studying their prevalence and tissue distribution in vivo in health, aging, and disease, and how they likely interact via their GPCR homo-, hetero-, and/or oligo-mers in order to know what implications that has for homeostasis, and for the progression of inflammation-based diseases such as PDD and cancer, where these receptors play such critical roles. That way, as Alekseenko et al. (2023) [114] suggest, we can, along the same lines, parlay “…the Sparkling Hope of Supramolecular Targeted Strategies” to give us the cures we need for inflammation-based diseases.

## Data Availability

No new data were created or analyzed as this is a review. Data sharing is not applicable to this article.

## References

[1] Gude D., Koduganti R.R., Prasanna S.J., Pothini L.R. (2012). Mouth: A portal to the body. Dent. Res. J..

[2] Weintraub J.A. (2022). The Oral Health in America Report: A Public Health Research Perspective. Prev. Chronic. Dis..

[3] Gill S.A., Steiner B. (2018). Oral Health Integration: A Call to Action. Fam. Med..

[4] Silk H., Savageau J.A., Sullivan K., Sawosik G., Wang M. (2018). An UpDate of Oral Health Curricula in US Family Medicine Residency Programs. Fam. Med..

[5] US Department of Health and Human Services (2000). Oral Health in America: A Report of the Surgeon General.

[6] Capodiferro S., Limongelli L., Favia G. (2021). Oral and Maxillo-Facial Manifestations of Systemic Diseases: An Overview. Medicina.

[7] Jin L.J., Lamster I.B., Greenspan J.S., Pitts N.B., Scully C., Warnakulasuriya S. (2016). Global burden of oral diseases: Emerging concepts, management and interplay with systemic health. Oral Dis..

[8] Hajishengallis G., Chavakis T. (2021). Local and systemic mechanisms linking periodontal disease and inflammatory comorbidities. Nat. Rev. Immunol..

[9] Preshaw P.M., Taylor J.J., Jaedicke K.M., De Jager M., Bikker J.W., Selten W., Bissett S.M., Whall K.M., van de Merwe R., Areibi A. (2020). Treatment of periodontitis reduces systemic inflammation in type 2 diabetes. J. Clin. Periodontol..

[10] Michaud D.S., Lu J., Peacock-Villada A.Y., Barber J.R., Joshu C.E., Prizment A.E., Beck J.D., Offenbacher S., Platz E.A. (2018). Periodontal Disease Assessed Using Clinical Dental Measurements and Cancer Risk in the ARIC Study. J. Natl. Cancer Inst..

[11] Michaud D.S., Kelsey K.T., Papathanasiou E., Genco C.A., Giovannucci E. (2016). Periodontal disease and risk of all cancers among male never smokers: An uPDDated analysis of the Health Professionals Follow-up Study. Ann. Oncol..

[12] Kim I.S., Yang W.S., Kim C.H. (2023). Physiological Properties, Functions, and Trends in the Matrix Metalloproteinase Inhibitors in Inflammation-Mediated Human Diseases. Curr. Med. Chem..

[13] Fields G.B. (2019). The Rebirth of Matrix Metalloproteinase Inhibitors: Moving Beyond the Dogma. Cells.

[14] Bertrand K.A., Shingala J., Evens A., Birmann B.M., Giovannucci E., Michaud D.S. (2017). Periodontal disease and risk of non-Hodgkin lymphoma in the Health Professionals Follow-Up Study. Int. J. Cancer.

[15] Fukushima N., Ishii S., Tsujiuchi T., Kagawa N., Katoh K. (2015). Comparative analyses of lysophosphatidic acid receptor-mediated signaling. Cell. Mol. Life Sci..

[16] Knowlden S., Georas S.N. (2014). The autotaxin-LPA axis emerges as a novel regulator of lymphocyte homing and inflammation. J. Immunol..

[17] Shea B.S., Tager A.M. (2012). Role of the lysophospholipid mediators lysophosphatidic acid and sphingosine 1-phosphate in lung fibrosis. Proc. Am. Thorac. Soc..

[18] Binder B.Y., Williams P.A., Silva E.A., Leach J.K. (2015). Lysophosphatidic Acid and Sphingosine-1-Phosphate: A Concise Review of Biological Function and Applications for Tissue Engineering. Tissue Eng. Part B Rev..

[19] Park C.H. (2019). Biomaterial-Based Approaches for Regeneration of Periodontal Ligament and Cementum Using 3D Platforms. Int. J. Mol. Sci..

[20] Hines O.J., Ryder N., Chu J., McFadden D. (2000). Lysophosphatidic acid stimulates intestinal restitution via cytoskeletal activation and remodeling. J. Surg. Res..

[21] Ridley A.J., Hall A. (1992). The small GTP-binding protein rho regulates the assembly of focal adhesions and actin stress fibers in response to growth factors. Cell.

[22] Ridley A.J., Hall A. (1994). Signal transduction pathways regulating Rho-mediated stress fibre formation: Requirement for a tyrosine kinase. EMBO J..

[23] Tomar A., George S.P., Mathew S., Khurana S. (2009). Differential effects of lysophosphatidic acid and phosphatidylinositol 4,5-bisphosphate on actin dynamics by direct association with the actin-binding protein villin. J. Biol. Chem..

[24] Dittmar T., Hass R. (2023). Intrinsic signalling factors associated with cancer cell-cell fusion. Cell Commun. Signal..

[25] Gupta G.P., Massagué J. (2006). Cancer metastasis: Building a framework. Cell.

[26] Hanahan D., Weinberg R.A. (2000). The hallmarks of cancer. Cell.

[27] Melzer C., von der Ohe J., Hass R. (2018). Enhanced metastatic capacity of breast cancer cells after interaction and hybrid formation with mesenchymal stroma/stem cells (MSC). Cell Commun. Signal..

[28] Melzer C., Ohe J.V., Hass R. (2020). Altered tumor plasticity after different cancer cell fusions with MSC. Int. J. Mol. Sci..

[29] Melzer C., von der Ohe J., Hass R. (2019). Involvement of Actin Cytoskeletal Components in Breast Cancer Cell Fusion with Human Mesenchymal Stroma/Stem-Like Cells. Int. J. Mol. Sci..

[30] Hass R. (2020). Role of MSC in the Tumor Microenvironment. Cancers.

[31] Cerutis D.R., Dreyer A., Cordini F., McVaney T.P., Mattson J.S., Parrish L.C., Romito L., Huebner G.R., Jabro M. (2004). Lysophosphatidic acid modulates the regenerative responses of human gingival fibroblasts and enhances the actions of platelet-derived growth factor. J. Periodontol..

[32] Cerutis D.R., Dreyer A.C., Vierra M.J., King J.P., Wagner D.J., Fimple J.L., Cordini F., McVaney T.P., Parrish L.C., Wilwerding T.M. (2007). Lysophosphatidic acid modulates the healing responses of human periodontal ligament fibroblasts and enhances the actions of platelet-derived growth factor. J. Periodontol..

[33] George J., Headen K.V., Ogunleye A.O., Perry G.A., Wilwerding T.M., Parrish L.C., McVaney T.P., Mattson J.S., Cerutis D.R. (2009). Lysophosphatidic Acid signals through specific lysophosphatidic Acid receptor subtypes to control key regenerative responses of human gingival and periodontal ligament fibroblasts. J. Periodontol..

[34] Cerutis D.R., Headen K.V., Perry G., Parrish L.C., McVaney T.P., Jordan C.S. (2010). Lysophosphatidic acid (LPA) receptor subtypes on human gingival and periodontal ligament fibroblasts are regulated by PDDGF. FASEB J..

[35] Lin C.I., Chen C.N., Lin P.W., Chang K.J., Hsieh F.J., Lee H. (2007). Lysophosphatidic acid regulates inflammation-related genes in human endothelial cells through LPA1 and LPA3. Biochem. Biophys. Res. Commun..

[36] Kihara Y., Mizuno H., Chun J. (2015). Lysophospholipid receptors in drug discovery. Exp. Cell Res..

[37] Yatomi Y., Ruan F., Hakomori S., Igarashi Y. (1995). Sphingosine-1-phosphate: A platelet-activating sphingolipid released from agonist-stimulated human platelets. Blood.

[38] Lin C.I., Chen C.N., Lin P.W., Lee H. (2007). Sphingosine 1-phosphate regulates inflammation-related genes in human endothelial cells through S1P1 and S1P3. Biochem. Biophys. Res. Commun..

[39] Hashimura S., Kido J., Matsuda R., Yokota M., Matsui H., Inoue-Fujiwara M., Inagaki Y., Hidaka M., Tanaka T., Tsutsumi T. (2020). A low level of lysophosphatidic acid in human gingival crevicular fluid from patients with periodontitis due to high soluble lysophospholipase activity: Its potential protective role on alveolar bone loss by periodontitis. BBA-Mol. Cell. Biol. Lipids.

[40] Bathena S.P., Huang J., Nunn M.E., Miyamoto T., Parrish L.C., Lang M.S., McVaney T.P., Toews M.L., Cerutis D.R., Alnouti Y. (2011). Quantitative determination of lysophosphatidic acids (LPAs) in human saliva and gingival crevicular fluid (GCF) by LC-MS/MS. J. Pharm. Biomed. Anal..

[41] Cerutis D.R., Weston M.D., Ogunleye A.O., McVaney T.P., Miyamoto T. (2014). Lysophosphatidic acid (LPA) 18:1 transcriptional regulation of primary human gingival fibroblasts. Genom Data.

[42] Cerutis D.R., Weston M.D., Alnouti Y., Bathena S.P., Nunn M.E., Ogunleye A.O., McVaney T.P., Headen K.V., Miyamoto T. (2015). A Major Human Oral Lysophosphatidic Acid Species, LPA 18:1, Regulates Novel Genes in Human Gingival Fibroblasts. J. Periodontol..

[43] Gruber R., Kandler B., Jindra C., Watzak G., Watzek G. (2004). Dental pulp fibroblasts contain target cells for lysophosphatidic Acid. J. Dent. Res..

[44] Thorlakson H.H., Engen S.A., Schreurs O., Schenck K., Blix I.J.S. (2017). Lysophosphatidic acid induces expression of genes in human oral keratinocytes involved in wound healing. Arch. Oral Biol..

[45] Kim D.H., Seo E.J., Tigyi G.J., Lee B.J., Jang I.H. (2020). The role of lysophosphatidic acid receptor 1 in inflammatory response induced by lip-opolysaccharide from Porphyromonas gingivalis in human periodontal ligament stem cells. International. J. Oral Biol..

[46] Vatėnas I., Linkevičius T. (2022). The use of the connective tissue graft from the palate for vertical soft tissue augmentation during submerged dental implant placement: A case series. Clin. Exp. Dent. Res..

[47] Liu Y., Karode K., Bodine P.V.N., Yaworsky P.J., Robinson J.A., Billiard J. (2010). LPA induces osteoblast differentiation through interplay of two receptors: LPA1 and LPA4. J. Cell. Biochem..

[48] Gennero I., Laurencin-Dalicieux S., Conte-Auriol F., Briand-Mésange F., Laurencin D., Rue J., Beton N., Malet N., Mus M., Tokumura A. (2011). Absence of the lysophosphatidic acid receptor LPA1 results in abnormal bone development and decreased bone mass. Bone.

[49] Chen X., Song Z., Chen R., Tan S., Huang C., Liu Y., Cheng B., Fu Q. (2019). Lysophosphatidic acid enhanced the osteogenic and angiogenic capability of osteoblasts via LPA1/3 receptor. Connect. Tissue Res..

[50] Tanaka M., Okudaira S., Kishi Y., Ohkawa R., Iseki S., Ota M., Noji S., Yatomi Y., Aoki J., Arai H. (2006). Autotaxin stabilizes blood vessels and is required for embryonic vasculature by producing lysophosphatidic acid. J. Biol. Chem..

[51] Van Meeteren L.A., Ruurs P., Stortelers C., Bouwman P., van Rooijen M.A., Pradère J.P., Pettit T.R., Wakelam M.J., Saulnier-Blache J.S., Mummery C.L. (2006). Autotaxin, a secreted lysophospholipase D, is essential for blood vessel formation during development. Mol. Cell Biol..

[52] Economides A.N., Frendewey D., Yang P., Dominguez M.G., Dore A.T., Lobov I.B., Persaud T., Rojas J., McClain J., Lengyel P. (2013). Conditionals by inversion provide a universal method for the generation of conditional alleles. Proc. Natl. Acad. Sci. USA.

[53] Ye X., Hama K., Contos J.J., Anliker B., Inoue A., Skinner M.K., Suzuki H., Amano T., Kennedy G., Arai H. (2005). LPA3-mediated lysophosphatidic acid signalling in embryo implantation and spacing. Nature.

[54] Hama K., Aoki J., Inoue A., Endo T., Amano T., Motoki R., Kanai M., Ye X., Chun J., Matsuki N. (2007). Embryo spacing and implantation timing are differentially regulated by LPA3-mediated lysophosphatidic acid signaling in mice. Biol. Reprod..

[55] Vittek J., Hernandez M.R., Wenk E.J., Rappaport S.C., Southren A.L. (1982). Specific estrogen receptors in human gingiva. J. Clin. Endocrinol. Metab..

[56] Mariotti A., Mawhinney M. (2013). Endocrinology of sex steroid hormones and cell dynamics in the periodontium. Periodontol 2000.

[57] Hwang Y.S., Lee S.K., Parke K., Chunga W. (2012). Secretion of IL-6 and IL-8 from lysophosphatidic acid-stimulated oral squamous cell carcinoma promotes osteoclastogenesis and bone resorption. Oral Oncol..

[58] Nakasaki T., Tanaka T., Okudaira S., Hirosawa M., Umemoto E., Otani K., Jin S., Bai Z., Hayasaka H., Fukui Y. (2008). Involvement of the lysophosphatidic acid-generating enzyme autotaxin in lymphocyte-endothelial cell interactions. Am. J. Pathol..

[59] Payne S.G., Milstien S., Spiegel S. (2002). Sphingosine-1-phosphate: Dual messenger functions. FEBS Lett..

[60] Xia P., Wadham C. (2011). Sphingosine 1-phosphate, a key mediator of the cytokine network: Juxtacrine signaling. Cytokine Growth Factor Rev..

[61] Hatoum D., Haddadi N., Lin Y., Nassif N.T., McGowan E.M. (2017). Mammalian sphingosine kinase (SphK) isoenzymes and isoform expression: Challenges for SphK as an oncotarget. Oncotarget.

[62] Blaho V.A., Hla T. (2014). An uPDDate on the biology of sphingosine 1-phosphate receptors. J. Lipid Res..

[63] Olivera A., Allende M.L., Proia R.L. (2013). Shaping the landscape: Metabolic regulation of S1P gradients. Biochim. Biophys. Acta..

[64] Moritz E., Wegner D., Groß S., Bahls M., Dörr M., Felix S.B., Ittermann T., Oswald S., Nauck M., Friedrich N. (2017). Reference intervals for serum sphingosine-1-phosphate in the population-based Study of Health in Pomerania. Clin. Chim. Acta.

[65] Moritz E., Wegner D., Groß S., Bahls M., Dörr M., Felix S.B., Ittermann T., Oswald S., Nauck M., Friedrich N. (2017). Data on subgroup specific baseline characteristics and serum sphingosine-1-phosphate concentrations in the Study of Health in Pomerania. Data Brief.

[66] Moritz E., Jedlitschky G., Negnal J., Tzvetkov M.V., Daum G., Dörr M., Felix S.B., Völzke H., Nauck M., Schwedhelm E. (2021). Increased Sphingosine-1-Phosphate Serum Concentrations in Subjects with Periodontitis: A Matter of Inflammation. J. Inflamm. Res..

[67] Rhim J.H., Jang I.S., Choi J.S., Kwon H.J., Yeo E.J., Park S.C. (2009). Time-dependent differential gene expression in lysophosphatidic acid-treated young and senescent human diploid fibroblasts. Mech. Ageing Dev..

[68] Luheshi N.M., Giles J.A., Lopez-Castejon G., Brough D. (2012). Sphingosine regulates the NLRP3-inflammasome and IL-1 beta release from macrophages. Eur. J. Immunol..

[69] Yang J., Yang L., Tian L., Ji X., Yang L., Li L. (2018). Sphingosine 1-phosphate (S1P)/S1P receptor 2/3 axis promotes inflammatory M1 polarization of bone marrow-derived monocyte/macrophage via G(α)i/o/PI3K/JNK pathway. Cell Physiol. Biochem..

[70] Kim B.J., Shin K.O., Kim H., Ahn S.H., Lee S.H., Seo C.H., Byun S.E., Chang J.S., Koh J.M., Lee Y.M. (2016). The effect of sphingosine-1-phosphate on bone metabolism in humans depends on its plasma/bone marrow gradient. J. Endocrinol. Invest..

[71] Bourgoin S.G., Zhao C. (2010). Autotaxin and lysophospholipids in rheumatoid arthritis. Curr. Opin. Investig. Drugs.

[72] Burg N., Salmon J.E., Hla T. (2022). Sphingosine 1-phosphate receptor-targeted therapeutics in rheumatic diseases. Nat. Rev. Rheumatol..

[73] Eskan M.A., Rose B.G., Benakanakere M.R., Lee M.J., Kinane D.F. (2008). Sphingosine 1-phosphate 1 and TLR4 mediate IFN-beta expression in human gingival epithelial cells. J. Immunol..

[74] Yu H., Sun C., Argraves K.M. (2016). Periodontal inflammation and alveolar bone loss induced by Aggregatibacter actinomycetemcomitans is attenuated in sphingosine kinase 1-deficient mice. J. Periodontal. Res..

[75] Meerschaert K., De Corte V., De Ville Y., Vandekerckhove J., Gettemans J. (1998). Gelsolin and functionally similar actin-binding proteins are regulated by lysophosphatidic acid. EMBO J..

[76] Wang Y., Bi X., Luo Z., Wang H., Ismtula D., Guo C. (2023). Gelsolin: A comprehensive pan-cancer analysis of potential prognosis, diagnostic, and immune biomarkers. Front. Genet..

[77] Thorp H.H., Yaffe M.B. (2023). Seeing is great, understanding is better. Sci. Signal..

[78] Xu Y. (2019). Targeting Lysophosphatidic Acid in Cancer: The Issues in Moving from Bench to Bedside. Cancers.

[79] Choi J.W., Herr D.R., Noguchi K., Yung Y.C., Lee C.W., Mutoh T., Lin M.E., Teo S.T., Park K.E., Mosley A.N. (2010). LPA receptors: Subtypes and biological actions. Annu. Rev. Pharmacol. Toxicol..

[80] Fujiwara Y., Sardar V., Tokumura A., Baker D., Murakami-Murofushi K., Parrill A., Tigyi G. (2005). Identification of residues responsible for ligand recognition and regioisomeric selectivity of lysophosphatidic acid receptors expressed in mammalian cells. J. Biol. Chem..

[81] Parrill A.L. (2008). Lysophospholipid interactions with protein targets. Biochim. Biophys. Acta.

[82] Im D.S., Heise C.E., Harding M.A., George S.R., O’Dowd B.F., Theodorescu D., Lynch K.R. (2000). Molecular cloning and characterization of a lysophosphatidic acid receptor, Edg-7, expressed in prostate. Mol. Pharmacol..

[83] Ray M., Nagai K., Kihara Y., Kussrow A., Kammer M.N., Frantz A., Bornhop D.J., Chun J. (2020). Unlabeled lysophosphatidic acid receptor binding in free solution as determined by a compensated interferometric reader. J. Lipid Res..

[84] Mizuno H., Kihara Y., Kussrow A., Chen A., Ray M., Rivera R., Bornhop D.J., Chun J. (2019). Lysophospholipid G protein-coupled receptor binding parameters as determined by backscattering interferometry. J. Lipid Res..

[85] Yanagida K., Masago K., Nakanishi H., Kihara Y., Hamano F., Tajima Y., Taguchi R., Shimizu T., Ishii S. (2009). Identification and characteri-zation of a novel lysophosphatidic acid receptor, p2y5/LPA6. J. Biol. Chem..

[86] Hannun Y., Obeid L. (2008). Principles of bioactive lipid signalling: Lessons from sphingolipids. Nat. Rev. Mol. Cell Biol..

[87] Leblanc R., Peyruchaud O. (2015). New insights into the autotaxin/LPA axis in cancer development and metastasis. Exp. Cell Res..

[88] Sugiura T., Nakane S., Kishimoto S., Waku K., Yoshioka Y., Tokumura A. (2002). Lysophosphatidic acid, a growth factor-like lipid, in the saliva. J. Lipid Res..

[89] Chae C.S., Sandoval T.A., Hwang S.M., Park E.S., Giovanelli P., Awasthi D., Salvagno C., Emmanuelli A., Tan C., Chaudhary V. (2022). Tumor-Derived Lysophosphatidic Acid Blunts Protective Type I Interferon Responses in Ovarian Cancer. Cancer Discov..

[90] Wang D., DuBois R.N. (2016). The Role of Prostaglandin E(2) in Tumor-Associated Immunosuppression. Trends Mol. Med..

[91] Bell C.R., Pelly V.S., Moeini A., Chiang S.C., Flanagan E., Bromley C.P., Clark C., Earnshaw C.H., Koufaki M.A., Bonavita E. (2022). Chemotherapy-induced COX-2 upregulation by cancer cells defines their inflammatory properties and limits the efficacy of chemoimmunotherapy combinations. Nat. Commun..

[92] Reinartz S., Lieber S., Pesek J., Brandt D.T., Asafova A., Finkernagel F., Watzer B., Nockher W.A., Nist A., Stiewe T. (2019). Cell type-selective pathways and clinical associations of lysophosphatidic acid biosynthesis and signaling in the ovarian cancer microenvironment. Mol. Oncol..

[93] Dacheux M.A., Lee S.C., Shin Y., Norman D.D., Lin K.H., E S., Yue J., Benyó Z., Tigyi G.J. (2022). Prometastatic Effect of ATX Derived from Alveolar Type II Pneumocytes and B16-F10 Melanoma Cells. Cancers.

[94] Turner J.A., Fredrickson M.A., D’Antonio M., Katsnelson E., MacBeth M., Van Gulick R., Chimed T.S., McCarter M., D’Alessandro A., Robinson W.A. (2023). Lysophosphatidic acid modulates CD8 T cell immunosurveillance and metabolism to impair anti-tumor immunity. Nat. Commun..

[95] Abdul Rahman M., Mohamad Haron D.E., Hollows R.J., Abdul Ghani Z.D.F., Ali Mohd M., Chai W.L., Ng C.C., Lye M.S., Karsani S.A., Yap L.F. (2020). Profiling lysophosphatidic acid levels in plasma from head and neck cancer patients. PeerJ..

[96] Cerutis D.R., Kumar D., Nichols M.G., Roemer G., Fluent M., Beller L., Miyamoto T., Alnouti Y. (2023). Lysophosphatidic Acid (LPA) Salivary Species Detection and In Situ LPAR Localization in the Intact Mouse Salivary Gland. J. Pharmacol. Exp. Ther..

[97] Goetzl E.J., Lee H., Azuma T., Stossel T.P., Turck C.W., Karliner J.S. (2000). Gelsolin binding and cellular presentation of lysophosphatidic acid. J Biol Chem..

[98] Watt M.J., Hoy A.J. (2012). Lipid metabolism in skeletal muscle: Generation of adaptive and maladaptive intracellular signals for cellular function. Am. J. Physiol. Endocrinol. Metab..

[99] Piktel E., Levental I., Durnaś B., Janmey P.A., Bucki R. (2018). Plasma Gelsolin: Indicator of Inflammation and Its Potential as a Diagnostic Tool and Therapeutic Target. Int. J. Mol. Sci..

[100] Feldt J., Schicht M., Garreis F., Welss J., Schneider U.W., Paulsen F. (2019). Structure, regulation and related diseases of the actin-binding protein gelsolin. Expert Rev Mol Med..

[101] Fleming J.K., Glass T.R., Lackie S.J., Wojciak J.M. (2016). A novel approach for measuring sphingosine-1-phosphate and lysophosphatidic acid binding to carrier proteins using monoclonal antibodies and the Kinetic Exclusion Assay. J. Lipid Res..

[102] Galvani S., Sanson M., Blaho V.A., Swendeman S.L., Obinata H., Conger H., Dahlbäck B., Kono M., Proia R.L., Smith J.D. (2015). HDL-bound sphingosine 1-phosphate acts as a biased agonist for the endothelial cell receptor S1P1 to limit vascular inflammation. Sci Signal..

[103] Blaho V.A., Galvani S., Engelbrecht E., Lin C., Swendeman S.L., Kono M., Proia R.L., Steinman L., Han M.H., Hla T. (2015). HDL-bound sphingosine-1-phosphate restrains lymphopoesis and neuroinflammation. Nature.

[104] Wilkerson B.A., Grass G.D., Wing S.B., Argraves W.S., Argraves K.M. (2012). Sphingosine-1-phosphate (S1P) carrier-dependent regulation of endothelial barrier: High density lipoprotein (HDL)-S1P prolongs endothelial barrier enhancement as compared with albumin-S1P via effects on levels, trafficking, and signaling of S1P1. J. Biol. Chem..

[105] Dahm F., Nocito A., Bielawska A., Lang K.S., Georgiev P., Asmis L.M., Bielawski J., Madon J., Hannun Y.A., Clavien P.A. (2006). Distribution and dynamic changes of sphingolipids in blood in response to platelet activation. J. Thromb. Haemost..

[106] Farooqui A.A., Horrocks L.A., Farooqui T. (2007). Interactions between neural membrane glycerophospholipid and sphingolipid mediators: A recipe for neural cell survival or suicide. J. Neurosci. Res..

[107] Gonzalez de San Roman E., Llorente-Ovejero A., Martinez-Gardeazabal J., Moreno-Rodriguez M., Gimenez-Llort L., Manuel I., Rodriguez-Puertas R. (2021). Modulation of neurolipid signaling and specific lipid species in the triple transgenic mouse model of Alzheimer’s disease. Int. J. Mol. Sci..

[108] Schwartz S.A., Reyzer M.L., Caprioli R.M. (2003). Direct tissue analysis using matrix-assisted laser desorption/ionization mass spectrometry: Practical aspects of sample preparation. J. Mass Spectrom..

[109] Wang J., Wang C., Han X. (2018). Enhanced coverage of lipid analysis and imaging by matrix-assisted laser desorption/ionization mass spectrometry via a strategy with an optimized mixture of matrices. Anal. Chim. Acta.

[110] Lipidomics Standards Initiative Consortium (2019). Lipidomics needs more standardization. Nat. Metab..

[111] Takeda H., Kawasaki A., Takahashi M., Yamada A., Koike T. (2003). Matrix-assisted laser desorption/ionization time-of-flight mass spectrometry of phosphorylated compounds using a novel phosphate capture molecule. Rapid Commun. Mass Spectrom..

[112] Morishige J., Urikura M., Takagi H., Hirano K., Koike T., Tanaka T., Satouchi K. (2010). A clean-up technology for the simultaneous determination of lysophosphatidic acid and sphingosine-1-phosphate by matrix-assisted laser desorption/ionization time-of-flight mass spectrometry using a phosphate-capture molecule, Phos-tag. Rapid Commun. Mass Spectrom..

[113] Iwama T., Kano K., Saigusa D., Ekroos K., van Echten-Deckert G., Vogt J., Aoki J. (2021). Development of an on-tissue derivatization method for MALDI mass spectrometry imaging of bioactive lipids containing phosphate monoester using Phos-tag. Anal. Chem..

[114] Alekseenko I., Kondratyeva L., Chernov I., Sverdlov E. (2023). From the catastrophic objective irreproducibility of cancer research and unavoidable failures of molecular targeted therapies to the sparkling hope of supramolecular targeted strategies. Int. J. Mol. Sci..

[115] Casadó V., Cortés A., Mallol J., Pérez-Capote K., Ferré S., Lluis C., Franco R., Canela E.I. (2009). GPCR homomers and heteromers: A better choice as targets for drug development than GPCR monomers?. Pharmacol. Ther..

[116] Gurevich V.V., Gurevich E.V. (2008). How and why do GPCRs dimerize?. Trends Pharmacol. Sci..

[117] Hazum E., Chang K.J., Cuatrecasas P. (1979). Opiate (Enkephalin) receptors of neuroblastoma cells: Occurrence in clusters on the cell surface. Science.

[118] Vischer H.F., Castro M., Pin J.P. (2015). G Protein-Coupled Receptor Multimers: A Question Still Open Despite the Use of Novel Ap-proaches. Mol. Pharmacol..

[119] Albizu L., Cottet M., Kralikova M., Stoev S., Seyer R., Brabet I., Roux T., Bazin H., Bourrier E., Lamarque L. (2010). Time-resolved FRET between GPCR ligands reveals oli-gomers in native tissues. Nat. Chem. Biol..

[120] Rivero-Müller A., Chou Y.Y., Ji I., Lajic S., Hanyaloglu A.C., Jonas K., Rahman N., Ji T.H., Huhtaniemi I. (2010). Rescue of defective G pro-tein-coupled receptor function in vivo by intermolecular cooperation. Proc. Natl. Acad. Sci. USA.

[121] Kasai R.S., Ito S.V., Awane R.M., Fujiwara T.K., Kusumi A. (2018). The Class-A GPCR Dopamine D2 Receptor Forms Transient Dimers Stabi-lized by Agonists: Detection by Single-Molecule Tracking. Cell Biochem. Biophys..

[122] Wang M., Pei L., Fletcher P.J., Kapur S., Seeman P., Liu F. (2010). Schizophrenia, amphetamine-induced sensitized state and acute ampheta-mine exposure all show a common alteration: Increased dopamine D2 receptor dimerization. Mol. Brain.

[123] Liu J., Tang H., Xu C., Zhou S., Zhu X., Li Y., Prézeau L., Xu T., Pin J.P., Rondard P. (2022). Biased signaling due to oligomerization of the G protein-coupled platelet-activating factor receptor. Nat. Commun..

[124] Zhu T., Gobeil F., Vazquez-Tello A., Leduc M., Rihakova L., Bossolasco M., Bkaily G., Peri K., Varma D.R., Orvoine R. (2006). In-tracrine signaling through lipid mediators and their cognate nuclear G-protein-coupled receptors: A paradigm based on PGE2, PAF, and LPA1 receptors. Can. J. Physiol. Pharmacol..

[125] Godbole A., Lyga S., Lohse M.J., Calebiro D. (2017). Internalized TSH receptors en route to the TGN induce local Gs-protein signaling and gene transcription. Nat. Commun..

[126] Cerutis D.R., Nichols M.G., Khan S.A., Miyamoto T. (2017). Localization of the Platelet-Activating Factor Receptor on the Intact Human Periodontal Ligament. FASEB J..

[127] Cerutis D.R., Nichols M., Hironaka M., Miyamoto T., Khan S., Ogunleye A., McVaney T. (2015). Complementing Confocal Detection of Antibody-labeled Lysophosphatidic Acid Receptors in Human Gingivae with Label-free Second Harmonic Generation Confocal Microscopy Detection of Collagen. FASEB J..

[128] Milstein J.N., Nino D.F., Zhou X., Gradinaru C.C. (2022). Single-molecule counting applied to the study of GPCR oligomerization. Biophys. J..

[129] Shonberg J., Scammells P.J., Capuano B. (2011). Design strategies for bivalent ligands targeting GPCRs. ChemMedChem.

[130] Lee S.C., Fujiwara Y., Tigyi G.J. (2015). Uncovering unique roles of LPA receptors in the tumor microenvironment. Recept. Clin. Investig..

[131] Rahaman M., Costello R.W., Belmonte K.E., Gendy S.S., Walsh M.T. (2006). Neutrophil sphingosine 1-phosphate and lysophosphatidic acid receptors in pneumonia. Am. J. Respir. Cell Mol. Biol..

[132] Panigrahy D., Gilligan M.M., Serhan C.N., Kashfi K. (2021). Resolution of inflammation: An organizing principle in biology and medicine. Pharmacol. Ther..

[133] Zaslavsky A., Singh L.S., Tan H., Ding H., Liang Z., Xu Y. (2006). Homo- and hetero-dimerization of LPA/S1P receptors, OGR1 and GPR4. Biochim. Biophys. Acta.

[134] Hisano Y., Kono M., Cartier A., Engelbrecht E., Kano K., Kawakami K., Xiong Y., Piao W., Galvani S., Yanagida K. (2019). Lysolipid receptor cross-talk regulates lymphatic endothelial junctions in lymph nodes. J. Exp. Med..

[135] GPCR Interaction Network. http://www.gpcr-hetnet.com/.

[136] Stoeber M., Jullié D., Lobingier B.T., Laeremans T., Steyaert J., Schiller P.W., Manglik A., von Zastrow M. (2018). A Genetically Encoded Biosensor Reveals Location Bias of Opioid Drug Action. Neuron.

[137] Shariati L., Esmaeili Y., Haghjooy Javanmard S., Bidram E., Amini A. (2021). Organoid technology: Current standing and future perspectives. Stem Cells.

[138] Zhao X., Xu Z., Xiao L., Shi T., Xiao H., Wang Y., Li Y., Xue F., Zeng W. (2021). Review on the vascularization of organoids and organoids-on-a-chip. Front. Bioeng. Biotechnol..

[139] Bhushan A., Senutovich N., Bale S.S., McCarty W.J., Hegde M., Jindal R., Golberg I., Usta O.B., Yarmush M.L., Vernetti L. (2013). Towards a three-dimensional microfluidic liver platform for predicting drug efficacy and toxicity in humans. Stem Cell Res. Ther..

[140] Hoffman B., Zinocker S., Holm S., Lewis J., Kavouras P. (2022). Organoids in the clinic: A systematic review of outcomes. Cells Tissues Organs.

